# Unusual presentation of PYGM gene mutation as late-onset McArdle disease with camptocormia: a case report

**DOI:** 10.1186/s13256-024-04802-x

**Published:** 2024-10-08

**Authors:** Johannes Stalter, Ursula Gies, Christian Mathys, Karsten Witt

**Affiliations:** 1 Department of Neurology, Carl Von Ossietzky University Oldenburg, Oldenburg, Germany; 2 University Clinic of Neurology, Evangelical Hospital Oldenburg, Oldenburg, Germany; 3 Section of Clinical Neuropathology, Klinikum Bremen Mitte, Bremen, Germany; 4 Institute of Radiology and Neuroradiology, Evangelical Hospital Oldenburg, University of Oldenburg, Oldenburg, Germany; 5 Research Center Neurosensory Science, University of Oldenburg, Oldenburg, Germany; 6 Department of Diagnostic and Interventional Radiology, University of Düsseldorf, Düsseldorf, Germany

**Keywords:** McArdle’s disease, Glycogen storage disease V, Camptocormia, Late-Onset

## Abstract

**Background:**

Glycogen storage disease type 5 (McArdle disease) leads to a deficiency in the activity of myophosphorylase resulting in an impaired glucose utilization. The disease can be caused by a variety of mutations in the PYGM gene, and its typical clinical manifestation is muscles weakness within the first three decades of life.

**Case presentation:**

In this case report we present the diagnostic work-up of a physically active 78-year-old Caucasian patient suffering from a 2-year history of progressive camptocormia including clinical, radiologic, histological, and genetic tests. There was no history of neuro-muscular diseases in the family. Serum CK levels were moderately increased while other blood/urine parameters were normal. Magnetic resonance imaging showed fatty remodeling of the muscles of the back. Histochemical examination of a muscle biopsy revealed the absence of myophosphorylase activity, while gene analysis identified a known early-onset McArdle mutation in the PYGM gene.

**Conclusion:**

This case highlights that the clinical spectrum of PYGM gene mutation typically manifest during adolescence, but it is also a differential diagnosis in late onset muscle disorders and emphases the investigation of the role of ACE inhibitors in this disease.

## Background

Glycogen storage disease type V (GSD-V), known as McArdle’s disease, is a rare genetic disorder that affects skeletal muscle metabolism, typically caused by a mutation in the PYGM gene [1, 2]. This leads to a deficiency in the enzyme myophosphorylase, which impairs the ability to utilize glucose from glycogen storage. Especially during exercise symptoms, such as fatigue, muscle cramping, and myoglobinuria, are evident. Signs and symptoms of this typical PYGM mutation usually are an early clinical manifestation demonstrating severe muscle weakness in the first three decades of life. About 150 mutations within the PYGM gene are known, but most of them seem to be very rare with a few mutations being responsible for the majority of cases [3, 4]. While the disease usually presents in childhood, in this case report, we present a patient who did not develop symptoms until late adulthood, and a bent trunk was the dominant symptom.

## Case description

**Fig. 1:**
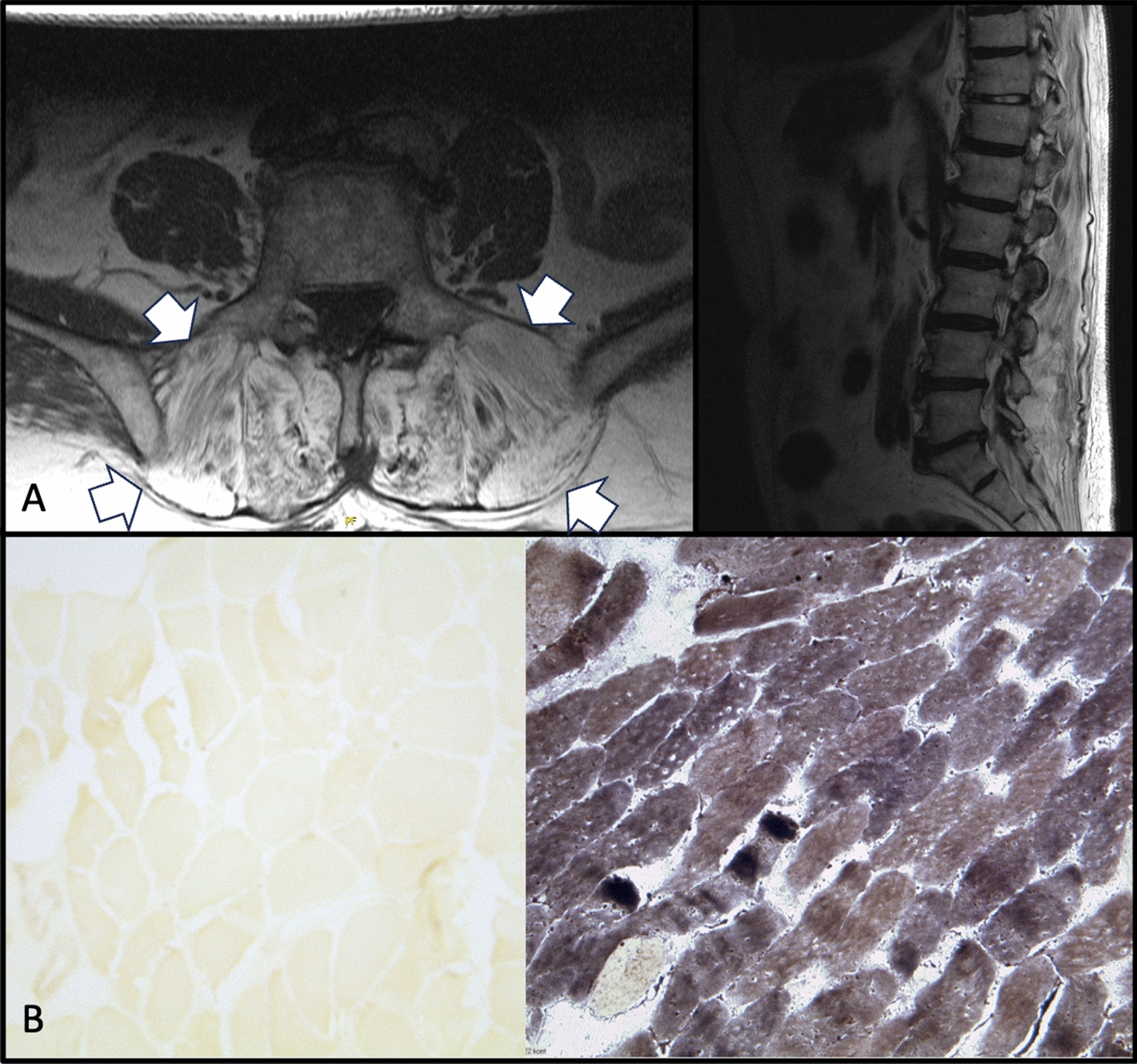
Magnetic resonance imaging scans demonstrate severe fatty degeneration of the autochthonous back muscles (white arrows) (T2w TSE transversal and sagittal. Siemens Symphony 1,5 T MRT) **A** Histochemical straining demonstrated the absence of myophosphorylase reaction in muscle fibres of the biopsy (left). Control sample with a normal myophosphorylase reaction in muscle fibres (right) (Left semitendinosus muscle, 10 × zoom, Histochemical enzyme reaction) (**B**). Written consent of the patient for publication of this photo was obtained

A 78-year-old Caucasian patient presented with a history of progressive gait impairment, camptocormia, and pain in his upper leg. He noticed a forward bending of the upper body, which started 2 years ago. There was no known family history of neurological or muscular disorders. The patient was physically active until the time of admission. Clinical examination revealed atrophy of the shoulder girdle with mild weakness of both shoulders and the left thigh. Achilles tendon reflex was absent. He showed a camptocormia (with a back-to-leg angle > 30°), which could not be actively compensated (Fig. 1). The further neurological status was unremarkable. Other diagnoses were hypertension treated with an AT1-inhibitor and generalized atherosclerosis treated with acetylsalicylic acid. Laboratory testing revealed elevated creatinine kinase (585 U/l, norm < 190 U/l). All other examined blood/urine parameters were normal. Electromyogenic (EMG) recordings of the lumbar autochthonic muscles revealed no electric activity whereas the lower proximal limbs (M. semitendinosus and M. biceps femoris) showed spontaneous activity, amplitude reduction, shortened muscle potentials, and decreased amplitude of the interference pattern as a myogenic signature. In the EMG, decreased amplitudes and moderate increased conduction times indicating a mild axonal-demyelinating sensorimotor polyneuropathy of the lower limbs were evident. Magnetic resonance imaging (MRI) scans of the back (Fig. 1A) showed severe fatty remodeling of the autochthonic muscles. The biopsy of the left semitendinosus muscle revealed atrophy of single muscular fibers, with increased storage of glycogen together with the total absence of myophosphorylase activity (for enzyme reaction see Fig. 1B) and sub-sarcolemmnal glycogen. Sanger sequencing of the PYGM gene (NM_005609.2) showed a heterozygous c.280 > T/p (Arg94Trp) and a heterozygous c.2056G > A/p (Gly686Arg) mutation compatible with early-onset McArdle’s disease. The current therapy consists of physiotherapy and the avoidance of potential muscle damaging drugs; with this regimen, the patient only shows a mild progression of the disease. Since the patient has no children, no genetic consultation has taken place.

## Discussion

In this case report, we report the history of a patient who showed initial signs of McArdle disease at the age of 76 years. Extensive testing revealed two causing heterozygous mutations in the PYGM gene. Comparing the clinical picture the patient presented, some striking features can be noticed. Besides muscle pain and very mild weakness of single muscles, the patient exhibited no other “classic” symptoms of McArdle disease. Our literature research revealed two cases with axial symptoms, such as camptocormia, in patients with McArdle disease [2, 3].

A register study found exercise intolerance in 99.5% of patients with McArdle disease [3]. This is even more noteworthy, since the muscle biopsy showed the total absence of myophosphorylase activity in this patient, e.g., a severe case of the disease. Despite these objective findings, the patient never experienced exercise intolerance, even though he was active throughout his life. An active lifestyle may favor a better clinical outcome; therefore, this might be a factor that prevented the emergence of earlier symptoms [3]. The same study found age to be negatively correlated with severe and progressive disease courses. Considering literature, the age of onset was very late. Only few cases are known where the disease presented after the first three decades of life [7]. Other case reports with the same mutations described likewise null myophosphorylase activity, but higher CK serum levels and early onset [8]. However, whether physical activity alone can explain those differences is contentious. It is known that a change in the ACE gene locus is associated with McArdle disease. A study investigated the effects of ACE-inhibitors in McArdle disease’s and found signs of a beneficial effect, although no long-term effect could be shown [9]. In summary, it could be possible that the intake of ACE-inhibitors for several years in combination with an active lifestyle by the described patient delayed the emergence of symptoms, which occurred right after the removal of the medication in this case.

## Conclusion

This case report shows the importance of considering rare genetic diseases, even with a late onset and an uncommon presentation. We highlight the problem that even though there are a lot of mutations known to cause McArdle, there is still no clear connection between the geno- and phenotype. The potential effect of ACE-inhibitors underlines the importance of further studies in this direction. This article therefore broadens the clinical and diagnostic picture of GSD-V.

## Data Availability

Data sharing is not applicable to this article as no datasets were generated or analyzed during the current study.
